# Progestin Primed Ovarian Stimulation (PPOS) protocol yields lower euploidy rate in older patients undergoing IVF

**DOI:** 10.1186/s12958-023-01124-3

**Published:** 2023-08-08

**Authors:** Angel Hsin-Yu Pai, Yen Ju Sung, Chia-Jung Li, Chieh- Yu Lin, Chia Lin Chang

**Affiliations:** Department of Obstetrics and Gynecology, Chang Gung Memorial Hospital Linkou Medical Center, Chang Gung University, 5 Fu-Shin Street, Kweishan, Taoyuan Taiwan

**Keywords:** Preimplantation genetic testing for aneuploidy (PGT-a), Progestin-primed ovarian stimulation (PPOS), GnRH-antagonist, Euploidy rate

## Abstract

**Background:**

To explore if exogenous progestin required for progestin primed ovarian stimulation (PPOS) protocol compromises the euploidy rate of patients who underwent preimplantation genetic testing cycles when compared to those who received the conventional gonadotropin-releasing hormone (GnRH) antagonist protocol.

**Methods:**

This retrospective cohort study analyzed 128 preimplantation genetic testing for aneuploidy (PGT-A) cycles performed from January 2018 to December 2021 in a single university hospital-affiliated fertility center. Infertile women aged 27 to 45 years old requiring PGT-A underwent either PPOS protocol or GnRH-antagonist protocol with in-vitro fertilization (IVF) or intracytoplasmic sperm injection (ICSI) for fertilization. Frozen embryo transfers were performed following each PGT-A cycle. Data regarding the two groups were analyzed using the Statistical Package for Social Sciences (SPSS) version 22.0 (SPSS Inc., Chicago, IL).

**Results:**

Patients who underwent PPOS treatment had significantly reduced blastocyst formation rate and euploidy rate compared to those who received the GnRH antagonist protocol. Subgroup-analysis was performed by stratifying patients’ age into elder and young subgroups (elder: ≥ 38-year-old, young: < 38-year-old). In the elder sub-population, the blastocyst formation rate of the PPOS group was significantly lower than that of the GnRH-antagonist group (45.8 ± 6.1% vs. 59.9 ± 3.8%, *p* = 0.036). Moreover, the euploidy rate of the PPOS group was only about 20% of that of  the GnRH-antagonist group (5.4% and 26.7%, *p* = 0.006). In contrast, no significant differences in blastocyst formation rate (63.5 ± 5.7% vs. 67.1 ± 3.2%, *p* = 0.45) or euploidy rate (30.1% vs. 38.5%, *p* = 0.221) were observed in the young sub-population. Secondary outcomes, which included implantation rate, biochemical pregnancy rate, clinical pregnancy rate, live birth rate, and miscarriage rate, were comparable between the two treatment groups, regardless of age.

**Conclusion:**

When compared to the conventional GnRH-antagonist approach, PPOS protocol could potentially reduce the euploidy rate in aging IVF patients. However, due to the retrospective nature of this study, the results are to be interpreted with caution. Before the PPOS protocol is widely implemented, further studies exploring its efficacy in larger populations are needed to define the optimal patient selection suitable for this method.

**Trial registration:**

Human Investigation and Ethical Committee of Chang Gung Medical Foundation (202200194B0).

## Background

Controlled ovarian hyperstimulation (COH), which involves the administration of exogenous gonadotropins to stimulate ovarian follicle growth, is a crucial checkpoint of the in vitro fertilization (IVF)/artificial reproductive technology (ART) procedure. During COH, the rise in estradiol level can sometime induce a premature luteinizing hormone (LH) surge and early release of oocytes that compromise the retrieval of a cohort of mature oocytes. To overcome this obstacle, various COH protocols have been established to suppress premature LH surge. Notably, the gonadotropin-releasing hormone antagonist (GnRH-antagonist) protocol is among the most widely used methods, because it effectively prevents premature LH surge [1, 2] and reduces the risk of ovarian hyperstimulation syndrome (OHSS) [3]. However, such protocol is associated with disadvantages like multiple injections, frequent monitoring and dose adjustment, and high therapeutic cost [4]. Recently, a newer stimulation regimen, progestin primed ovarian stimulation (PPOS), has emerged with the goal of preventing premature LH surge in a more patient-friendly manner [5].

The PPOS protocol uses exogenous progesterone to suppress endogenous LH secretion in the early follicular phase and maintain a stable hormonal environment for the induction of final oocyte maturation. Earlier studies have shown that the clinical outcomes of PPOS procedures are comparable to that of conventional methods [6–8]. Owing to its ease in drug administration, PPOS protocol has gained popularity as the more flexible and patient-friendly COH approach [9–11]. Although PPOS is effective, there are concerns about the potential side effects of progestin stimulation on oocyte competence, embryo availability, embryo implantation, and obstetric outcome, especially for patients who have difficulties achieving a successful IVF outcome. In addition, the PPOS protocol has been reported to yield a lower number of retrieved oocytes [12] and exert negative influences on granular cell functions [13–16] and follicle growths [17]. In some patient populations, the PPOS protocol could even result in a lower cumulative live birth rate and higher cycle cancellation rate when compared to the GnRH-antagonist protocol [18].

In this retrospective study, we seek to clarify whether the PPOS protocol has an adverse impact on oocyte competence and clinical outcomes when compared to the conventional GnRH-antagonist protocol in a cohort of patients who received PGT-A cycles. The results of this study indicate that the PPOS protocol may negatively impact the embryo quality in elder patients, and age may be a critical factor in determining its utility.

## Materials and methods

### Study subjects

This retrospective study included infertile patients who received PGT-A cycles for advanced maternal age (46.9%, 60/128), recurrent miscarriage (12.5%, 16/128), recurrent IVF failure (22.6%, 29/128), severe male infertility (0.8%, 1/128), or personal request regarding the chromosomal status of the embryos (17.2%, 22/128) from January 2018 to December 2021 in the Chang Gung Memorial Hospital Fertility Center.

Exclusion criteria included those with body mass index (BMI) greater than 30 kg/m^2^, known chromosomal translocation, endocrine disorders, systemic diseases, and Mullerian malformations. Baseline characteristics, such as the main cause of infertility and ovarian reserve status, were not subjected to exclusion. The study included 128 patients, with an average age of 37.5 years old, categorized into the PPOS arm (34 patients) and the conventional GnRH-antagonist arm (94 patients) (Table 1). The number of previous IVF treatment failure (averaged 1.8 for PPOS and 1.9 for GnRH-antagonist, *p* = 0.58) and previous miscarriage (averaged 1.1 for PPOS and 0.8 for GnRH-antagonist, *p* = 0.09) were comparable between the two arms. Choices of ovarian stimulation protocol and gonadotropin dosage were adjusted based on the patient's age, BMI, hormone levels, antral follicle count (AFC), and prior responses to ovarian stimulation. The study was reviewed and approved by the institutional review board of the Human Investigation and Ethical Committee of Chang Gung Medical Foundation (202200194B0).

**Table 1:** Patients’ demographic characteristics and IVF cycle-related variables of the PPOS and GnRH antagonist treatment groups

		**All patients**			**Age < 38**			**Age ≥ 38**	
	**PPOS *N* = 34 (cycles)**	**Antagonist *N* = 94 (cycles)**	***P* Value**	**PPOS *N* = 15 (cycles)**	**Antagonist *N* = 47 (cycles)**	***P* Value**	**PPOS *N* = 19 (cycles)**	**Antagonist *N* = 47 (cycles)**	***P* Value**
Age (years)	37.9 ± 4.5	37.3 ± 4.4	0.540	33.4 ± 2.2	33.8 ± 2.9	0.656	41.1 ± 1.9	40.9 ± 2.0	0.350
BMI (kg/m^2^)	23.14 ± 3.86	22.59 ± 3.09	0.410	22.78 ± 4.4	22.63 ± 2.83	0.877	23.42 ± 3.49	22.55 ± 3.35	0.350
Infertility years	3.2 ± 2.2	3.5 ± 2.0	0.468	2.1 ± 0.9	3.4 ± 1.9	0.019	4.1 ± 2.5	3.7 ± 2.1	0.503
AMH (ng/ml)	2.56 ± 1.83	2.56 ± 2.1	0.986	3.056 ± 2.206	2.905 ± 2.351	0.839	2.166 ± 1.420	2.299 ± 1.871	0.790
Gonadotropin dose (IU)	2269.06 ± 768.93	2200.72 ± 545.05	0.664	1417.60 ± 954.12	2204.59 ± 643.19	0.0022	2596.54 ± 340.45	2197.44 ± 454.61	0.006
Day of trigger	10.9 ± 1.7	9.3 ± 1.2	< 0.001	11.1 ± 1.9	9.4 ± 1.2	< 0.001	10.8 ± 1.5	9.3 ± 1.2	< 0.001
Day 3 FSH (IU/L)	7.54 ± 2.76	8.08 ± 2.78	0.330	7.15 ± 2.33	8.42 ± 3.17	0.162	7.84 ± 3.09	7.76 ± 2.33	0.911
Day 3 LH (IU/L)	4.68 ± 2.14	4.7 ± 2.36	0.968	5.15 ± 1.92	4.50 ± 2.21	0.311	4.31 ± 2.28	4.90 ± 2.52	0.381
LH on trigger day (IU/L)	4.79 ± 4.02	4.48 ± 3.59	0.689	4.40 ± 4.70	4.54 ± 3.81	0.923	5.08 ± 3.45	4.42 ± 3.39	0.494
E2 on trigger day (pg/ml)	2314.9 ± 1956.5	1505.8 ± 1166.2	0.008	2630.4 ± 1692.0	1526.0 ± 1067.6	0.006	2065.9 ± 2154.7	1484.4 ± 1276.3	0.210
Oocytes retrieved	12.2 ± 8.3	12 ± 8.9	0.928	16.1 ± 9.7	12.3 ± 10.2	0.214	9.1 ± 5.6	11.7 ± 7.5	0.176
MII rate (%)	86.6 ± 2.7*	80.0 ± 1.7*	0.004	87.4 ± 3.7*	78.0 ± 2.8*	0.075	89.5 ± 3.8*	81.9 ± 2.1*	0.024
Fertilization rate (%)	79.0 ± 5.3*	76.4 ± 2.6*	0.832	87.1 ± 9.0*	80.4 ± 4.2*	0.463	72.7 ± 6.1*	72.5 ± 3.0*	0.975
Blastocyst rate (%)	53.6 ± 4.5*	63.5 ± 2.5*	0.042	63.5 ± 5.7*	67.1 ± 3.2*	0.454	45.8 ± 6.1*	59.9 ± 3.8*	0.036
Euploidy rate (%)	17.0 ± 4.6*	31.8 ± 3.5*	0.027	27.4 ± 6.2*	39.4 ± 5.4*	0.245	8.3 ± 6.1*	24.0 ± 4.2*	0.003

Data are expressed as mean ± SD or frequency (%)

^*^ mean ± standard error of the means (SEM)

*BMI* Body mass index, *AMH*  Anti-Müllerian hormone

*FSH*  Follicle stimulating hormone, *LH* Luteinizing hormone, *E*2 Estradiol, *M*II Metaphase II

### Treatment protocols

Starting on the second or third day of the menstrual cycle, individualized doses of gonadotropins, between 150–300 IU per day, were typically used, which included the following types of medications: recombinant-follicle-stimulating hormone (r-FSH) combined with recombinant-luteinizing hormone (r-LH, Pergoveris®, 150 IU/75 IU/vial, Merck Serono, Switzerland), recombinant-follicle-stimulating hormone (r-FSH, Gonal-F®, 5.5 mcg/vial, Merck Serono, Switzerland), human menopausal gonadotrophin (HMG, Menopur®, 75 IU/75 IU/vial, Ferring, Germany), or long-acting r-FSH (Elonva® 100 μg/0.5 ml or 150 µg/0.5 ml, Vetter Pharma-Fertigung, Germany).

On the fifth or sixth day of stimulation, follicular size and hormone levels were measured with laboratory and sonographic assessment, and the doses of gonadotropins would be adjusted accordingly. Either oral synthetic progestins or subcutaneous injections of a GnRH antagonist was used for the inhibition of premature LH surge.

In the PPOS arm, medical suppression would begin around day 3 of the menstrual cycle until the trigger day, with a daily dose of medroxyprogesterone acetate (MPA) tablet (Medrone, 10 mg, U-liang, Taiwan) or twice daily dose of dydrogesterone film-coated tablet (Duphaston, 10 mg, Abbott, Netherlands). Patients in the conventional GnRH-antagonist arm received an injection of cetrorelix acetate (Cetrotide®, 0.25 mg/vial, Merck Serono, Switzerland) daily, starting from the fifth day of ovarian stimulation until the trigger day. Final oocyte maturation was induced with 0.2 mg of triptorelin acetate (Decapeptyl, 0.1 mg/ml, Ferring, Germany) or 250 mcg of choriogonadotropin alfa (Ovidrel, 250 mcg, Merck Serono Italy), once the leading follicle reached 18 mm in diameter or greater. Transvaginal oocyte pick-up was performed 36–38 h later.

### Oocyte insemination, embryo culture and biopsy preimplantation genetic analysis

Retrieved oocytes were washed with fertilization medium (Sydney fertilization medium, COOK) and incubated in a 6% CO_2_, 5% O_2_, and 89% N_2_ 37 °C dry benchtop incubator (G210, K-Systems) for approximately two hours prior to the removal of cumulus cells. Oocytes were transferred to a medium with 40 ~ 120 IU/ml of recombinant human hyaluronidase (ICSI Cumulase, Origio) with a micropipette (Stripper; 275-μm inner diameter, Origio). Subsequent aspiration of the oocytes in and out of a micropipette (Flexipet; 140-μm inner diameters, Cook) multiple times completed the denuding process.

Around four hours after retrieval, intracytoplasmic sperm injection (ICSI) was performed on denuded, metaphase II oocytes. Bathed in culture medium (Continuous Single Culture-NX Complete, Irvine Scientific) under paraffin oil (OVOIL, Vitrolife), the inseminated oocytes were incubated in a 6% CO_2_, 5% O_2_, and 89% N_2_ dry benchtop incubator. Medium change-over (Continuous Single Culture-NX Complete, Irvine Scientific) took place on day 1 and day 3 of sequential culture. Fertilized embryos were observed and evaluated daily. Laserassisted hatching was performed on morula stage embryos, and blastocysts were graded with Gardner Classification prior to biopsy of the trophectoderm. Only blastocysts with a score of AA, AB, BA, BB, or BC were biopsied, which involved mechanical excision of 8–10 trophectoderm cells. The biopsied cells were aspirated with a biopsy pipette (Biopsy Pipette 15,115, 25-μm inner diameter, Vitrolife), washed twice with sterile 1 × phosphate-buffered saline solution (PBS-20X #9808, Cell Signaling), and centrifuged immediately. Cell pellets were stored at -20 °C prior to transporting to a reference laboratory for genetic analysis using next generation sequencing methods.

### Clinical outcomes and statistical analysis

The primary outcome evaluated euploidy rate while secondary outcomes included oocyte fertilization rate, blastocyst formation rate, number of oocytes retrieved, biochemical pregnancy rate, clinical pregnancy rate, live birth rate (per cycle), and miscarriage rate. To investigate the potential influence of maternal age, age-stratified subgroup analysis was performed by categorizing patients into those older than or equal to 38 years old or less than 38 years old.

Fertilization rate was calculated by taking the total number of 2 pronuclei stage zygotes divided by the total number of MII oocytes. Euploidy rate was defined as the total number of embryos proven to be euploid divided by the total number of embryos that were biopsied.

Data were analyzed using the Statistical Package for Social Sciences (SPSS) version 22.0 (SPSS Inc., Chicago, IL). The Kruskal–Wallis test was used to compare parameters between groups with non-normal distributions. The Mann–Whitney U test was applied to identify the group causing the difference. For qualitative data, X^2^ test was implemented while Student's *t*-test was used for small sample size sets. Results were presented as mean ± standard deviation or mean ± SEM, and a *p*-value of < 0.05 was considered statistically significant.

## Results

A total of 128 infertile women were included in this study, with 94 patients in the conventional GnRH-antagonist protocol and 34 patients in the PPOS protocol treatment groups (Table 1). The average age of the PPOS and GnRH-antagonist groups were 37.9 ± 4.5 and 37.3 ± 4.4 years, respectively. Baseline characteristics, such as age, body mass index (BMI), years of infertility, AMH level, were comparable between the two treatment groups. A total of 407 blastocysts derived from 1527 oocytes were obtained and eligible for analysis (Table 2). In both groups, there were no reported cases of severe OHSS or incidences of premature LH surge.

**Table 2:** The number of oocytes and blastocysts obtained as well as the euploidy rate per embryo following COH treatments

**Embryos from**		**All patients**			**Age < 38**			**Age ≥ 38**	
	**PPOS**	**Antagonist**	***P* Value**	**PPOS**	**Antagonist**	***P* Value**	**PPOS**	**Antagonist**	***P* Value**
Total No. of retrieved oocytes (N)	398	1129	N/A	216	580	N/A	182	549	N/A
Total No. of injected MII oocytes (N)	364	890	N/A	208	455	N/A	156	435	N/A
No. of 2PN (N)	273	645	N/A	156	333	N/A	117	312	N/A
No. of blastocysts (N)	110	291	N/A	73	156	N/A	37	135	N/A
No. euploid blastocysts (N)	24	96	N/A	22	60	N/A	2	36	N/A
Euploid blastocyst per injected MII (%)	6.5%	10.7%	N/A	10.5%	13.1%	N/A	1.3%	8.3%	N/A
Fertilization rate per MI oocytes (%)	273/364 (75%)	645/890 (72.5%)	0.605	156/208 (75.0%)	333/455 (73.2%)	0.622	117/156 (75.0%)	312/435 (71.7%)	0.431
Euploidy rate per blastocysts (%)	24/110 (26.8%)	96/291 (33.0%)	0.029	22/73 (30.1%)	60/156 (38.5%)	0.221	2/37 (5.4%)	36/135 (26.7%)	0.006

Data are expressed as mean ± SD or frequency (%)

Analysis of the entire study population showed that the total gonadotropin dose, FSH and LH levels on Day 3, and the LH level on trigger day were comparable between the treatment groups (Table 1). On the other hand, the day of trigger (10.9 ± 1.7 vs. 9.3 ± 1.2, *p* < 0.001) and the E2 level on trigger day (2314.9 ± 1956.5 vs. 1505.8 ± 1166.2, *p* = 0.008) were significantly greater in the PPOS group when compared to that of the GnRH-antagonist group.

While the number of oocytes retrieved and the fertilization rate of the two groups were similar, the percentage of MII oocytes in the PPOS group was significantly higher than that of the GnRH-antagonist group (86.6% vs. 80.0%, *p* = 0.004). However, patients in the PPOS group had significantly lower blastocyst formation rate (53.6 ± 4.5% vs. 63.5 ± 2.5%, *p* = 0.042) and euploidy rate (17.0 ± 4.6% vs. 31.8 ± 3.5%, *p* = 0.027) when compared to the GnRH-antagonist group. Additionally, analysis focused on a per embryo basis showed that the euploidy rate of PPOS group remains significantly lower than that of the GnRH-antagonist group (26.8% vs. 33.0%, *p* = 0.029) (Table 2), suggesting that the progestin protocol could affect the developmental processes of retrieved oocytes after fertilization.

Evaluation of clinical outcomes showed that after transfer of an euploid embryo or euploid embryos in either group results in similar clinical outcomes (Table 3). There were no significant differences in implantation rate (36.7% vs. 39.8%, *p* = 0.766), biochemical pregnancy rate (55% vs. 53%, *p* = 0.897), clinical pregnancy rate (40% vs. 41.7% *p* = 0.896), live birth rate (35% vs. 33.3%, *p* = 0.891), or miscarriage rate (5% vs. 8.3% *p* = 0.624) between the treatment groups (Table 3).

**Table 3:** Clinical outcomes after COH treatments

		**All patients**			**Age < 38**			**Age ≥ 38**	
**Cycles**	**PPOS *N* = 20**	**Antagonist *N* = 60**	***P* Value**	**PPOS *N* = 10**	**Antagonist *N* = 29**	***P* Value**	**PPOS *N* = 10**	**Antagonist *N* = 31**	***P*** **Value**
No. of embryo /ET cycle (N)	1.5 ± 0.8	1.4 ± 0.6	0.698	1.5 ± 0.7	1.4 ± 0.6	0.842	1.5 ± 1.0	1.4 ± 0.7	0.832
Biochemical pregnancy ( %)	55% (11/20)	53% (32/60)	0.897	10% (1/10)	10.3% (3/29)	0.975	0% (0/10)	3.2% (1/31)	0.565
Clinical Pregnancy ( %)	40% (8/20)	41.7% (25/60)	0.896	50% (5/10)	55.2% (16/29)	0.777	50% (5/10)	41.9% (13/31)	0.655
Implantation rate ( %)	36.7% (11/30)	39.8% (33/83)	0.766	33.3% (5/15)	46.3% (19/41)	0.384	35.7% (5/14)	33.3% (14/42)	0.871
Miscarriage rate ( %)	5% (1/20)	8.3% (5/60)	0.624	10% (1/10)	13.8% (4/29)	0.757	30% (3/10)	12.9% (4/31)	0.212
Live birth rate ( %)	35% (7/20)	33.3% (20/60)	0.891	40% (4/10)	41.4% (12/29)	0.939	20% (2/10)	29% (9/31)	0.575

Data are expressed as mean ± SD or frequency (%)

Sub-analysis by stratifying patients into young (< 38 years old) and elder (≥ 38 years old or older) revealed that the overall pattern of clinical differences between the treatment groups was only apparent in the elder subgroup (Tables 1 and 2). For patients ≥ 38 years old, those in the PPOS group (mean age = 41.1 ± 1.9) had significantly lower blastocyst formation rate (45.8 ± 6.1% vs. 59.9 ± 3.8%, *p* = 0.036) and euploidy rate (8.3 ± 6.1% vs. 24 ± 4.2%, *p* = 0.003) when compared to those in the GnRH-antagonist group (mean age = 40.9 ± 2.0) (Table 1). Likewise, when the data was analyzed on a per embryo basis, the euploidy rate of the PPOS group remained significantly lower than that of the GnRH-antagonist group (5.4% vs. 26.7%, *p* = 0.006) (Table 2). The rate of euploid blastocyst per injected MII oocyte of the elder PPOS and elder GnRH antagonist subgroup was 1.3% and 8.3%, respectively. The elder PPOS subgroup was triggered at a later day (10.8 ± 1.5 vs. 9.3 ± 1.2, *p* < 0.001) and had a significantly higher rate of MII formation (89.5% vs. 81.9%, *p* = 0.024) compared to the elder GnRH-antagonist subgroup (Table 1).

In contrast, there were no significant differences in blastocyst formation rate or euploidy rate between the young PPOS and young GnRH-antagonist subgroups (Table 1). Similarly, no significant difference in the euploidy rate was observed between the young subgroups when the data was analyzed on a per embryo basis (30.1% vs. 38.5%, *P* = 0.221) (Table 2). The rate of euploid blastocyst per injected MII oocyte of the young PPOS and young GnRH-antagonist subgroup was 10.5% and 13.1%, respectively. While patients in the young PPOS subgroup were also triggered later (day 11.1 ± 1.9 vs. day 9.4 ± 1.2, *p* < 0.001) when compared to those of the young GnRH-antagonist subgroup, they had a significantly higher level of E2 on the day of trigger (2630.4 ± 1692.0 vs. 1526.0 ± 1067.6, *p* = 0.006) compared to patients in the young GnRH-antagonist subgroup (Table 1).

 Similar to the results from the overall study population, there were no significant differences in implantation rate (36.7% vs 39.8% *p* = 0.766), biochemical pregnancy rate (55% vs 53% *p* = 0.897), clinical pregnancy rate (40% vs 41.7% *p* = 0.896), live birth rate (35% vs 33.3% *p* = 0.891), and miscarriage rate (5% vs. 8.3% *p* = 0.624) between the protocols in both the elder and young subgroups (Table 3).

## Discussion

The current retrospective analysis showed that while clinical outcomes were comparable, regardless of whether the patients received the PPOS or GnRH-antagonist protocol, there were significant differences in the blastocyst formation rate and euploidy rate between the two treatment groups in the older subset of patients. It is possible that: (1) the PPOS protocol is not be as effective as the GnRH-antagonist protocol for patients who are prone to aneuploidy formation, and (2) progestins mainly impact the quality of oocytes and embryos during early embryo formation but not oocyte formation or post-implantation development. Our results, furthermore, raised the question of whether, in elder patients, PPOS protocol is truly noninferior to the standard GnRH-antagonist protocol, in terms of embryo quality.

To prevent premature LH surge during COH, GnRH agonists like leuprolide were initially used to suppress the hypothalamic-pituitary-ovarian (HPO) axis prior to ovarian hyperstimulation. However, such practice occasionally led to prolonged suppression and reduced number of oocytes retrieved [19]. Meanwhile, the use of GnRH antagonists, such as ganirelix or cetrorelix, appeared to allow better control of ovulation and oocyte yield. While the application of GnRH-antagonist protocol had become increasingly prevalent [20], concerns about its cumbersome administration and risk of premature LH surge and OHSS remained [21].

In recent years, an alternative protocol that utilizes synthetic progestin to prevent premature ovulation has emerged and evolved into the PPOS protocol. Similar to the corpus luteum-derived progesterone, high doses of synthetic progestin, such as medroxyprogesterone 17-acetate (MPA), during COH can suppress LH level and prevent premature ovulation [1, 6, 22]. Studies in the past few years had shown that the use of PPOS and GnRH-antagonist protocols yielded comparable number of mature oocytes, blastocyst formation rate, number of good-quality embryos, implantation rate, clinical pregnancy rate, and live birth rate [23]. A meta-analysis further demonstrated that the PPOS protocol resulted in higher number of retrieved oocytes, MII oocytes, viable embryos, as well as a lower rate of premature LH surge than the control protocol in patients with diminished ovarian reserve or normal ovarian reserve [24]. Furthermore, the PPOS protocol seemed to lower the risk of OHSS when compared to the conventional methods, especially in patients with polycystic ovarian syndrome (PCOS) [25].

Because progestins can be administered orally, the PPOS protocol is considered more patient-friendly and has gained popularity and clinical advocates since 2015 [24]. A major short-coming, however, comes from progestin’s negative impact on endometrial receptivity [1], so such application requires freeze-all and FET cycles. As such, it has been proposed that PPOS should be the first choice for cases that require fertility preservation, not suitable for fresh embryo transfers (e.g., oocyte donors, PGT-A, and PGT-M cycles), or at high risk for OHSS.

However, since high levels of progestins had been shown to affect some aspects of ovarian physiology, we hypothesized that the PPOS protocol could impact specific steps of embryogenesis. Consistent with this theory, we noticed that while the PPOS protocol produced clinical outcomes comparable to that of the conventional protocol, embryos from the PPOS group had reduced euploidy and blastocyst rates. As euploidy rate is a crucial determinant of a successful IVF and is strongly correlated with maternal age, we further analyzed the data in age-stratified subgroups to factor in the influences of age. The age of 38 was used as the cutoff to divide the study population, because euploidy rate drastically declined after that age [26]. Importantly, we found that the difference in euploidy rate between the two treatment groups could be largely attributed to the results of the elder sub-population. These data suggested that, in selected patients, the use of PPOS protocol could significantly reduce the success of early embryo development but have negligible effect on oocyte yield under COH condition or post-implantation development [11, 25, 27, 28].

While the PPOS protocol's effects on diminished euploidy rate in the elder patients remain to be elucidated, we speculate that multiple factors were at play. First, it could be the result of local effects from supra-physiological progesterone. Not to mention, synthetic progestins exerted effects different from that of natural progesterone, owing to their distinct pharmacodynamic characteristics [29]. With unique affinities not just to progesterone receptors, synthetic progestins also act on other steroid receptors, such as androgen and mineralocorticoid receptors. Exposure of pharmacological progesterone had been shown to significantly reduce blastocyst formation rate in bovine cumulus–oocyte complexes [30] and inhibit the resumption of meiosis in mouse oocytes [31, 32]. Additionally, periovulatory exposure to high doses of progesterone could reduce the number of antral follicles and ovulation rate in hamsters, rabbits, and monkeys [33–35] and negatively impact oocyte health, meiosis, cytoplasmic maturation, and fertilization in nonhuman primates [36].

Likewise, earlier clinical studies of IVF patients had insinuated possible negative influences of elevated progesterone level on embryo quality [37–40]. A reduced cumulative live birth rate (CLBR) [39] and top-quality embryo formation rate [38, 40] were observed in cases with elevated serum progesterone on triggering day [39]. In addition, in a study with oocyte donation cycles, the PPOS protocol significantly reduced the biochemical pregnancy rate, clinical pregnancy rate, and live birth rate when compared to the use of conventional GnRH antagonist despite a comparable number of mature oocytes were retrieved [41]. Furthermore, the use of PPOS protocol had been consistently shown to associate with longer time to live birth (TTLB) in both unselected women [17] and patients of Poseidon Group 1 [42]. As such, pharmacological level of progestin required for the PPOS protocol could contribute to the observed reduction of euploidy rate.

Second, our results indicated that age could be the main contributing factor for the negative impact of PPOS on euploidy rate. While a recent study reported that PPOS had no impact on embryo quality [43], the discordant finding could be associated with the characteristics of patient populations. In the previous study, the average age of the patients was 37 years old, with 39 being the highest age . In contrast, 44.5% (57/128) of the patients in our study was ≥ 39 years old. The average age of elder PPOS and GnRH-antagonist subgroups was 41.1 ± 1.9 years and 40.9 ± 2.0 years, respectively. The lack of impact on euploidy rate in the prior study was more consistent with our observation of the young IVF patients. In addition, the difference in years of infertility between the populations of these studies could partly contribute to the dissimilarities in the results. While the prior study reported an average of 28–30 months of infertility, the elder patients in the present study experienced infertility for around 3.7–4.1 years. It is plausible that the damaging effects of progestins on embryo quality was exacerbated by the longer duration of infertility and the more advanced age. Taken together, these results imply that the PPOS protocol may reduce euploidy rate in aging patients due to (1) an altered hormonal environment that affects the maturation process and chromosomal integrity of early embryos; and (2) age- and infertility duration (i.e., years of infertility)-related decline in follicular environment.

Finally, it is important to note that our finding is unique because prior studies focused on other components of COH protocols and demonstrated that the dose of gonadotropins [44, 45], follicular phase progesterone level prior to oocyte retrieval [46], and the methods of LH suppression have negligible impacts on euploidy rate in IVF patients. Our results revealed that althoughy the PPOS protocol is a clinically acceptable method,  given its comparable clinical outcomes in the general population, it may have an impact on embryogenesis of oocytes for aging women. Therefore, cautions should be taken for whom or how a COH protocol is selected.

Major limitations of our study included the retrospective, non-randomized design and small sample size, which caused differences in baseline characteristics of the two treatment groups. Additionally, the lack of standardization in the initial dose and type of medications prescribed could have affected the final outcomes. Moreover, due to the chemical structure of dydrogesterone, we could not accurately monitor the progestin levels with simple blood tests. Overall, a study with a larger sample size could provide more robust results.

## Conclusion

In summary, our study indicates that the PPOS protocol yields a euploidy rate four times lower than that of the conventional GnRH-antagonist protocol for infertile patients aged 38 and above. While earlier studies that compared PPOS with other protocols have produced conflicting results, our study suggests that age may be an important factor to consider. Despite the advantages of a reduced cost and convenient drug administration, the application of PPOS protocol in aging IVF patients should be carefully reviewed to ensure favorable obstetrical and neonatal outcomes. Further studies regarding the efficacy of the PPOS protocol in a larger population are needed to help elucidate the optimal patient population suitable for this method before it is widely implemented.

## Data Availability

The datasets used and/or analyzed during the current study are available from the corresponding author on reasonable request.

## Abbreviations

AMH: Anti-mullerian hormone
ART: Artificial reproductive technology
BMI: Body mass index
COH: Controlled ovarian hyperstimulation
E2: Estradiol
FSH: Follicle-stimulating hormone
GnRH: Gonadotropin-releasing hormone
HMG: Human menopausal gonadotrophin
HPO: Hypothalamic-pituitary-ovarian
ICSI: Intracytoplasmic sperm injection
IVF: In-vitro fertilization
LH: Luteinizing hormone
MII: Metaphase II
OHSS: Ovarian hyperstimulation syndrome
PCOS: Polycystic ovarian syndrome
PGT-A: Preimplantation genetic testing for aneuploidy
PPOS: Progestin primed ovarian stimulation
r-FSH: Recombinant-follicle-stimulating hormone
r-LH: Recombinant-luteinizing hormone
